# Clinical characteristics and prognosis of newly diagnosed multiple myeloma patients with hypercalcemia: a retrospective cohort study

**DOI:** 10.3389/fmed.2026.1900926

**Published:** 2026-07-29

**Authors:** Lin Gui, Lili Yang, Xuan Zhou, Yang Chen, Zhinan Yang, Yongchao Ma, Baoan Chen, Wenjing Zhang

**Affiliations:** 1 Department of Hematology, The Affiliated Jiangning Hospital of Nanjing Medical University, Nanjing, Jiangsu, China; 2 Department of Hematology, Zhongda Hospital Southeast University, Nanjing, Jiangsu, China

**Keywords:** ferritin, hypercalcemia, minimal residual disease, multiple myeloma, prognosis

## Abstract

**Objective:**

To investigate the clinical characteristics and identify prognostic factors in patients with newly diagnosed multiple myeloma (NDMM) who presenting with hypercalcemia.

**Methods:**

We retrospectively analyzed clinical and laboratory data from 82 NDMM patients with hypercalcemia who were admitted to the Department of Hematology at Zhongda Hospital (Southeast University) and Jiangning Hospital (Nanjing Medical University) between January 2015 and December 2024. 80 patients without hypercalcemia were randomly selected as a control group for comparison. Prognostic evaluations were based on overall survival (OS), progression-free survival (PFS), and minimal residual disease (MRD) status. Survival rates were estimated using the Kaplan–Meier method, with univariate analyses performed via the Log-rank test and multivariate analyses utilizing the Cox proportional hazards regression model.

**Results:**

The cohort comprised 82 patients (51 males, 31 females). By the end of the follow-up period, with the median follow-up time was 51 months (range, 6–128): 59 patients had died and 23 were alive. Univariate analysis revealed that cytogenetic abnormalities (detected by FISH), serum creatinine, ferritin, MRD status, and serum calcium levels significantly affected both OS and PFS. Multivariate Cox regression demonstrated that the presence of ≤ 1 FISH abnormality and MRD negativity were independent protective factors for OS. Notably, elevated serum ferritin (≥500 ng/mL), a marker previously underexplored in this context, emerged as a robust independent risk factor for both OS and PFS. Severe hypercalcemia (≥3.5 mmol/L) was also identified as an independent risk factor for both OS and PFS. For PFS, MRD negativity remained an independent protective factor, whereas ferritin ≥500 ng/mL and serum calcium ≥3.5 mmol/L were identified as independent risk factors.

**Conclusion:**

NDMM patients with hypercalcemia exhibited a substantial tumor burden, heterogeneous clinical manifestations, and a high rate of early mortality. Prompt correction of serum calcium levels, with adjustment for concurrent hypoalbuminemia, is imperative. Early detection and optimal management of hypercalcemia are essential to refining treatment strategies and improving survival outcomes.

## 1 Introduction

Multiple myeloma (MM) is a hematological malignancy characterized by the clonal proliferation of plasma cells in the bone marrow, frequently accompanied by end-organ damage manifesting as anemia, hypercalcemia, renal impairment, and osteolytic lesions (collectively termed the “CRAB” criteria). Among these complications, hypercalcemia occurs in approximately 15–21% of patients with newly diagnosed MM (NDMM) (1, 2). It is primarily driven by increased bone resorption mediated by osteoclasts activation via the receptor activator of nuclear factor-κB (RANK)/RANK ligand (RANKL) pathway, as well as by the secretion of osteoclast-activating factors such as interleukin-6, macrophage inflammatory protein-1α, and tumor necrosis factor-α (3).

Clinically, hypercalcemia in MM is not merely a metabolic derangement but a critical emergency that can lead to a series of life-threatening complications. It impairs renal concentrating ability, resulting in polyuria, volume depletion, and prerenal azotemia, and promotes calcium salt deposition in the renal tubules, which may progress to nephrocalcinosis and acute kidney injury (AKI) (4, 5). Furthermore, severe hypercalcemia (commonly defined as serum calcium ≥3.5 mmol/L) can induce neuropsychiatric symptoms ranging from confusion and lethargy to coma, in addition to cardiac arrhythmias and gastrointestinal dysmotility (6–8). Therefore, prompt recognition and aggressive management of hypercalcemia are imperative for preventing irreversible organ damage and early mortality.

Despite its clinical significance, the prognostic implications of hypercalcemia in NDMM patients remain incompletely characterized. Existing studies have indicated that patients who present with hypercalcemia at initial diagnosis tend to exhibit higher tumor burden characteristics, such as advanced Durie-Salmon (DS) and International Staging System (ISS) stages, extensive osteolytic lesions, and a higher prevalence of renal dysfunction (9). However, most previous investigations have either included hypercalcemia as one of several prognostic factors in unselected MM cohorts or were limited by small sample sizes and a lack of long-term follow-up. Systematic clinical data focusing exclusively on the hypercalcemic subtype-particularly regarding its unique clinical presentation, treatment response, and independent prognostic factors remain scarce.

Moreover, the identification of reliable and readily measurable biomarkers to refine risk stratification in this high-risk subgroup remains an unmet clinical need. Although serum calcium and creatinine levels are routinely assessed, they may not fully capture the complex biology of aggressive MM. Ferritin, an acute-phase reactant and intracellular iron storage protein, has emerged as a potential prognostic marker in various hematological malignancies, including lymphoma and leukemia (10, 11). Elevated ferritin levels often reflect increased inflammatory burden, tumor activity, and macrophage activation, and they have been associated with poor outcomes in several cancers. In MM, hyperferritinemia has been reported in a subset of patients, particularly those with aggressive disease features, however, its prognostic value, especially in the context of hypercalcemia, has not been systematically evaluated (12).

Therefore, this study aimed to: (1) retrospectively analyze the clinical characteristics, laboratory parameters, and survival outcomes of 82 NDMM patients with hypercalcemia from two tertiary hospitals over a 10-year period; (2) identify independent prognostic factors for overall survival (OS) and progression-free survival (PFS) in this specific population; and (3) test the hypothesis that elevated serum ferririn might serve as an additional, independent prognosticator in this high-risk subgroup, beyond classic parameters such as calcium and renal function. Our findings are expected to provide a practical reference for the early identification of high-risk patients and the optimization of treatment strategies in NDMM patients who present with hypercalcemia.

## 2 Methods

### 2.1 Study population and data collection

Patients with newly diagnosed multiple myeloma and hypercalcemia who were admitted to the Department of Hematology of Zhongda Hospital affiliated with Southeast University, and the Department of Hematology at Jiangning Hospital, affiliated with Nanjing Medical University, from January 1, 2015 to December 31, 2024, were enrolled. All diagnoses met the International Myeloma Working Group (IMWG) criteria. Durie-Salmon (DS) and International Staging System (ISS) staging was performed according to the Chinese Guidelines for the Diagnosis and Treatment of Multiple Myeloma (10). This study was approved by the Ethics Committee of Jiangning Hospital (Approval No: 2026-03-006-K01), and written informed consent was obtained from all enrolled patients.

### 2.2 Indicators monitored

Demographic data, clinical manifestations, and routine blood parameters (white blood cell count, hemoglobin, and platelet count) were recorded at initial diagnosis. Biochemical parameters (albumin, globulin, creatinine, blood urea nitrogen, serum calcium, β2-microglobulin, and lactate dehydrogenase), light chain type, ferritin levels, bone marrow morphology, cytogenetic analysis (chromosome karyotyping and fluorescence *in situ* hybridization [FISH]), clinical stage (DS and ISS), and MRD status were also recorded. Hypercalcemia was defined as a corrected serum calcium level > 2.6 mmol/L. For patients with concurrent hypoalbuminemia (serum albumin < 30 g/L), corrected calcium was calculated using the following formula: corrected calcium (mmol/L) = measured total serum calcium (mmol/L) + 0.02 × (40 – measured serum albumin (g/L) (8). For patients with normal albumin levels, measured total calcium was used directly. Bone destruction was confirmed by MRI or PET-CT, and extramedullary disease (EMD) was verified by imaging, histopathology, and/or flow cytometry. Minimal residual disease (MRD) was assessed in patients who achieved at least a very good partial response (VGPR) after completion of induction therapy or autologous stem cell transplantation. Bone marrow MRD was detected using EuroFlow standardized secondary flow cytometry (NGF). The threshold for MRD detection in this study was set at 1 × 10^−5^; MRD negativity was defined as a proportion of clonal plasma cells accounting for < 0.001% of all nucleated bone marrow cells.

### 2.3 Treatment regimens and efficacy evaluation

Treatment modalities, including induction chemotherapy, autologous stem cell transplantation (ASCT), and maintenance therapy, along with their corresponding responses, were recorded. The induction treatment regimens include PD (Bortezomib and dexamethasone), PAD (Bortezomib, doxorubicin and dexamethasone), PCD (Bortezomib, cyclophosphamide and dexamethasone), VRD (Bortezomib, lenalidomide and dexamethasone.), PDD (Bortezomib, liposomal doxorubicin and dexamethasone) and ID (Ixazomib, and dexamethasone). Efficacy was evaluated according to the IMWG criteria and categorized as complete response (CR), very good partial response (VGPR), partial response (PR), minimal response (MR), stable disease (SD), and progressive disease (PD). The objective response rate (ORR) was defined as the proportion of patients achieving PR or better. Overall survival (OS) was calculated from the date of diagnosis to death or the last follow-up. Progression-free survival (PFS) was defined as the time from the initiation of induction therapy to disease progression, death, or the last follow-up. Disease progression in this study was defined as either MRD negativity followed by reversion to positivity the emergence of new lesions, or worsening of target organ damage.

### 2.4 Statistical analysis

Statistical analyses were performed using SPSS version 27.0 (IBM Corp., Armonk, NY, USA) and GraphPad Prism version 10.1.2 (GraphPad Software, Boston, MA, USA). Patient characteristics were summarized using descriptive statistics. Count data were compared using the χ^2^ test; Survival analyses were conducted using the Kaplan–Meier method, with group comparisons evaluated via the Log-rank test. Multivariate analysis was performed using the Cox proportional hazards regression model. A *P* value < 0.05 was considered statistically significant. For cytogenetic abnormalities detected by FISH, the cut-off of >1 abnormality vs. ≤ 1 abnormality was pre-specified based on the distribution of abnormality counts in our cohort (median number of abnormalities: 1; interquartile range: 1–2). This cut-off has previously been reported as prognostically relevant in MM populations.

## 3 Results

### 3.1 Clinical characteristics

From January 1, 2015, to December 31, 2024, a total of 767 patients were newly diagnosed with MM at the Department of Hematology, Zhongda Hospital (affiliated with Southeast University), and Jiangning Hospital (affiliated with Nanjing Medical University). Among them, 82 patients were diagnosed with concurrent hypercalcemia and were included in this study. The primary clinical symptoms of 82 patients included fatigue (46.3%), bone pain (25.7%), and respiratory (8.5%), digestive (7.3%), renal (6.1%), and neurological manifestations (6.1%). Among the six patients with gastrointestinal symptoms, three had vomiting, two had diarrhea, and one had gastrointestinal bleeding. The 5 patients with neurological symptoms included two with personality and behavioral changes, one with unilateral limb numbness, one with speech disorder, and one with headache. Patients with MM and hypercalcemia had more advanced DS and ISS stages, a higher incidence of renal insufficiency, elevated ferritin and β2-microglobulin levels, a higher incidence of FISH genetic abnormalities, and a lower MRD negative rate. FISH testing revealed cytogenetic abnormalities in 77 patients, while 5 were entirely negative. Detected abnormalities included 1q21 amplification (79.1%), 13p14 deletion (34.2%), IgH translocation (15.3%), 13q-(33.1%), 16q-(14.2%), 17p-(16.3%), t (4;14) (18.3%), t (11;14) (19.6%), and TP53 mutations (9.8%). Thirty-four patients harbored two or more abnormalities, 43 had a single abnormality, and nine exhibited complex karyotypes. All patients received bortezomib-based induction regimens, including PD, PAD, PCD, VRD, and PDD. Nine patients who achieved CR or VGPR proceeded to ASCT for consolidation. Maintenance therapy comprised lenalidomide-based regimens for 36 patients, while the remaining surviving patients received PD, ID and PCD (Table 1).

**Table 1:** Comparison of basic clinical characteristics between newly diagnosed MM patients with hypercalcemia and non-hypercalcemic MM patients.

Clinical features	High calcium group *N* (%)	Non-high calcium group *N* (%)	χ^2^	*P*
Age (years)			1.620	0.132
≥65	28 (34.1)	29 (36.3)		
< 65	54 (65.9)	51 (63.7)		
Sex			0.082	0.774
Male	51 (62.2)	48 (60)		
Female	31 (37.8)	32 (40)		
Myeloma type			1.567	0.321
IgG	33 (40.2)	35 (43.8)		
IgA	22 (26.8)	24 (30)		
IgD	4 (4.9)	4 (5.0)		
IgM	1 (1.3)	0 (0)		
Light	17 (20.7)	15 (18.6)		
Nonsecretory	2 (2.4)	1 (1.3)		
Biclone	3 (3.7)	1 (1.3)		
DS stage			6.213	0.022
I	3 (3.8)	10 (12.4)		
II	10 (12.2)	37 (46.3)		
III	69 (84.1)	33 (41.3)		
ISS stage			5.783	0.013
I	6 (7.5)	14 (17.5)		
II	18 (22.5)	38 (47.5)		
III	58 (66.6)	28 (35)		
HGB (g/L)			0.690	0.406
≥70	60 (73.2)	63 (78.8)		
< 70	22 (52.4)	17 (21.2)		
PLT (10^9^/L)			0.011	0.916
≥100	57 (69.5)	55 (68.8)		
< 100	25 (30.5)	25 (31.2)		
Cr (μmol/L)			6.604	0.014
≥177	32 (39)	17 (21.3)		
< 177	50 (61)	63 (78.7)		
ALB (g/L)			0.380	0.537
≥35	59 (72)	54 (67.5)		
< 35	23 (28)	26 (32.5)		
Ferritin (ng/mL)			15.126	< 0.01
≥500	39 (47.6)	15 (18.8)		
< 500	43 (52.4)	65 (81.2)		
EMD			1.098	0.137
With EMD	8 (9.8)	6 (7.5)		
Without EMD	74 (90.2)	74 (92.5)		
Prognosis			6.943	0.002
Death	59 (72)	38 (47.5)	7.285	0.016
Survival	23 (28)	42 (52.5)		
β2MG (mg/L)			3.679	0.046
≥3	78 (95.1)	68 (85)		
< 3	4 (4.9)	12 (15)		
MRD			6.954	0.001
MRD^−^	38 (46.3)	56 (70)		
MRD^+^	44 (53.7)	24 (30)		
FISH			5.672	0.023
Normal	5 (6.1)	15 (18.3)		
Abnormal	77 (93.9)	65 (81.7)		
PD	25 (30.5)	22 (27.5)	0.194	0.675
PAD	8 (9.8)	7 (8.7)	0.0488	0.825
PCD	14 (17.1)	13 (16.3)	0.011	0.915
VRD	23 (28.0)	25 (31.3)	0.199	0.656
PDD	5 (6.1)	6 (7.5)	0.029	0.864
ID	7 (8.5)	7 (8.7)	0.024	0.877

### 3.2 Response to treatment strategies

The median follow-up time was 51 months (range, 6, 128). Thirteen patients died early (within 3 months): three from gastrointestinal bleeding during the first cycle of PD, six from severe infections, one from sudden heart failure, and three from rapid disease progression. Among the 69 patients who completed ≥4 cycles of chemotherapy and underwent efficacy evaluation, the responses were: CR (*n* = 12), VGPR (*n* = 24), PR (*n* = 25), MR (*n* = 4), PD (*n* = 13), and SD (*n* = 4). The overall response rate (≥PR) was 88.4% (61/69 patients), and 38 patients achieved MRD negativity.

### 3.3 Univariate analysis of prognostic factors

**Figure 1:**
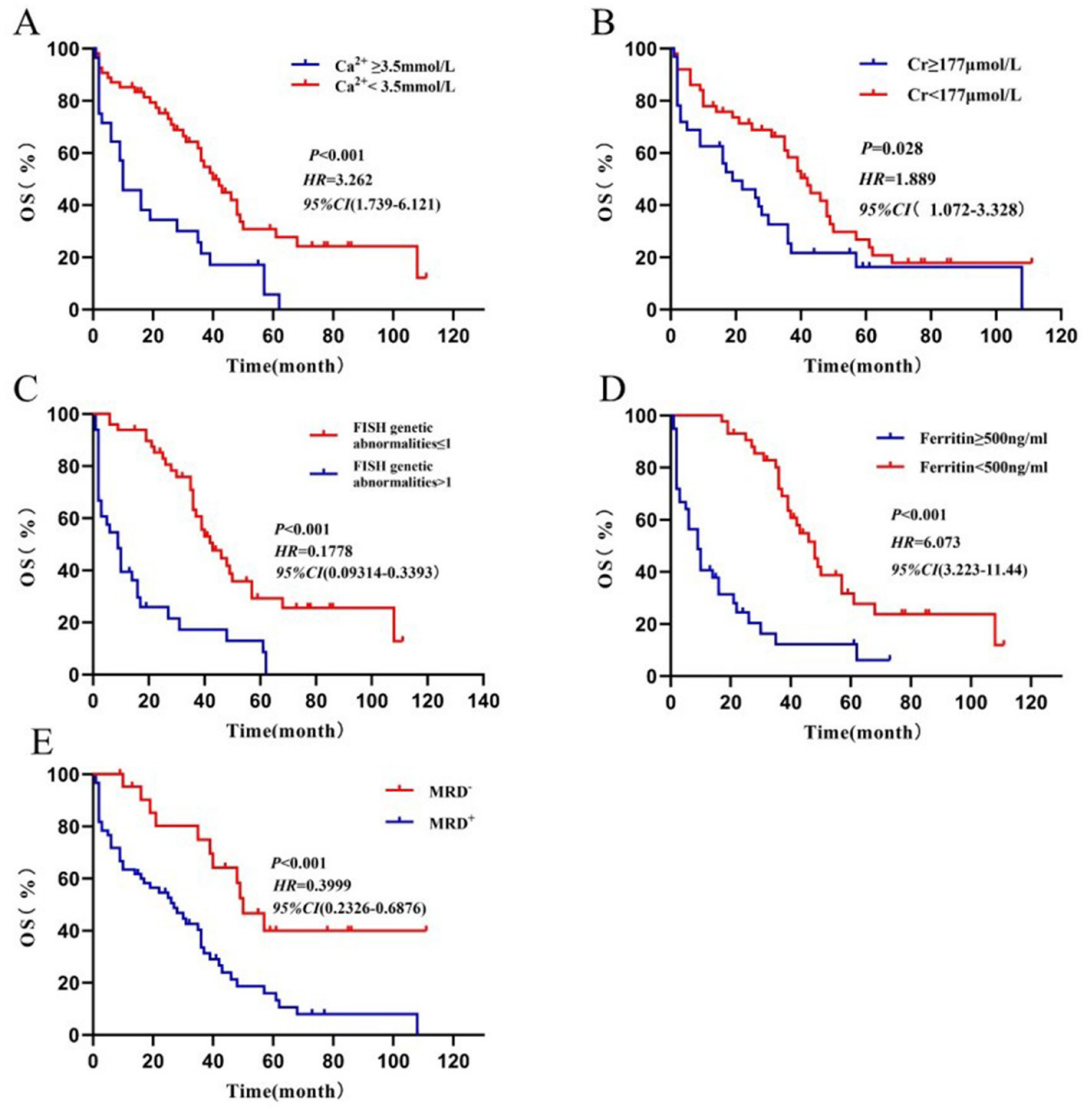
Kaplan–Meier curves for overall survival (OS) stratified by prognostic factors in NDMM patients with hypercalcemia. **(A)** Serum calcium (< 3.5 vs. ≥3.5 mmol/L); **(B)** serum creatinine (< 177 vs. ≥177 μmol/L); **(C)** number of FISH abnormalities ( ≤ 1 vs. >1); **(D)** serum ferritin (< 500 vs. ≥500 ng/mL); **(E)** MRD status (negative vs. positive). *P*-values were calculated using the log-rank test. Numbers at risk are shown below each x-axis. Abbreviations: NDMM, newly diagnosed multiple myeloma; FISH, fluorescence *in situ* hybridization; MRD, minimal residual disease.

**Figure 2:**
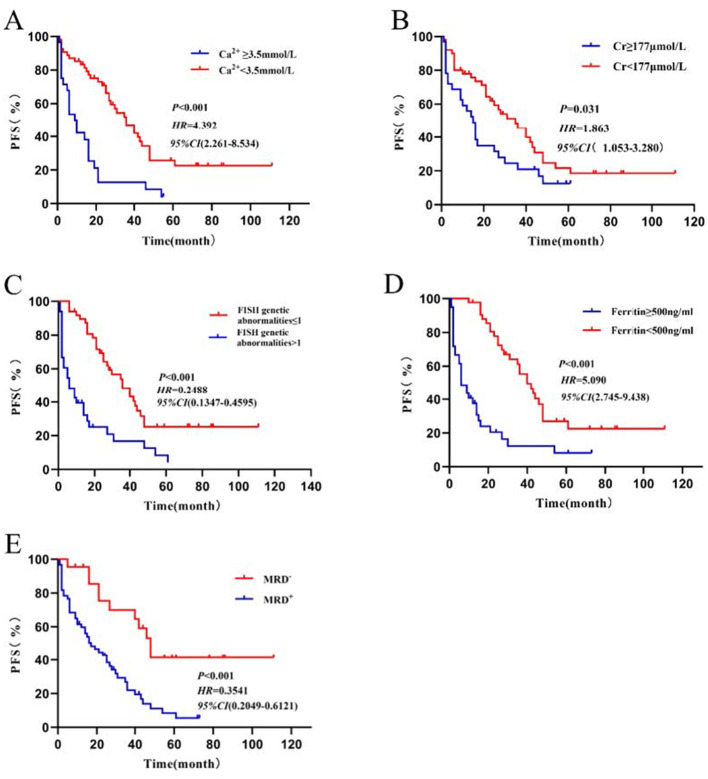
Univariate analysis of factors influencing progression-free survival in newly diagnosed multiple myeloma patients with hypercalcemia. **(A)** Effect of calcium on progression-free survival; **(B)** Effect of Cr on progression-free survival; **(C)** Effect of FISH on progression-free survival; **(D)** Effect of ferritin on progression-free survival; **(E)** Effect of MRD on progression-free survival.

The median overall survival (OS) for the entire cohort was 18.5 months (95% CI: 14.2–22, 8 months), and the median progression-free survival (PFS) was 12.3 months (95% CI: 9.1–15.5 months). Univariate analysis assessed the impact of demographic factors, laboratory parameters (hemoglobin, platelet count, creatinine, albumin, calcium, and ferritin), cytogenetics (FISH abnormalities > 1 vs. ≤ 1), treatment modalities (ASCT, and lenalidomide maintenance), and MRD status on OS and PFS. The results indicated that serum calcium, creatinine, ferritin, MRD status, and FISH results significantly affected both OS (*P* < 0.05, Figure 1) and PFS (*P* < 0.05, Figure 2).

### 3.4 Multivariate analysis of prognostic factors

Factors showing significance in the univariate analysis were incorporated into a Cox multivariate regression model. The results demonstrated that the presence of ≤ 1 FISH abnormality and MRD negativity were independent protective factors for OS, whereas ferritin ≥500 ng/mL and serum calcium ≥3.5 mmol/L were independent risk factors (Table 2). For PFS, MRD negativity served as an independent protective factor, whereas ferritin ≥500 ng/mL and serum calcium ≥3.5 mmol/L were independent risk factors (Table 3).

**Table 2:** Multivariate survival analysis of overall survival in newly diagnosed multiple myeloma patients with hypercalcemia.

Parameters	β	SE	Wald	*P*	HR	95% CI
FISH	0.841	0.303	7.726	0.005	0.431	0.238–0.780
MRD^**−**^	0.962	0.340	7.999	0.005	0.382	0.196–0.744
Fer ≥ 500	1.036	0.308	11.348	< 0.001	2.818	1.542–5.150
Cr ≥ 177	0.323	0.272	1.411	0.235	1.381	0.811–2.354
Ca ≥ 3.5	0.655	0.278	5.551	0.018	1.925	1.116–3.319

**Table 3:** Multivariate survival analysis of progression-free survival in newly diagnosed multiple myeloma patients with hypercalcemia.

Parameters	β	SE	Wald	*P*	HR	95% CI
MRD	0.980	0.346	8.006	0.005	0.375	0.190–0.740
Ca ≥ 3.5	0.793	0.289	7.520	0.006	2.211	1.254–3.897
Fer ≥ 500	0.927	0.292	10.086	0.001	2.526	1.426–4.476
FISH	−0.430	0.324	1.758	0.185	0.651	0.345–1.228
Cr ≥ 177	0.512	0.279	3.380	0.066	1.669	0.967–2.880

## 4 Discussion

Hypercalcemia is a critical metabolic emergency and a severe complication in multiple myeloma, with an incidence rate of 20–40% (13). Approximately one-third of MM patients present with hypercalcemia as their initial symptom, and another third develop it during disease progression (14).

Total serum calcium consists of ionized and protein-bound calcium. Albumin (ALB), the primary transport protein for blood calcium, significantly influences total calcium levels. Because routine biochemical assays measure total calcium, concurrent hypoalbuminemia—common in MM patients—can result in falsely depressed calcium readings. Therefore, adjustment of calcium levels according to ALB concentrations is crucial for avoiding underestimation of the true prevalence and severity of hypercalcemia (15, 16).

Clinically, hypercalcemia can induce profound neuropsychiatric symptoms (insomnia, confusion, coma), cardiac arrhythmias, and gastrointestinal disturbances (6, 17). Furthermore, it severely compromises renal concentrating ability and promotes calcium salt deposition, leading to nephrolithiasis and acute kidney injury (AKI) (18). In our cohort, five patients initially presented with neurological deficits, and severe cases rapidly deteriorated to coma or early death. Beacuse many patients initially present with nonspecific symptoms such as bone pain or fatigue, hypercalcemia can easily be overlooked.

NDMM patients with hypercalcemia typically exhibit a highly aggressive disease phenotype and elevated tumor burden and frequently present at advanced DS or ISS stages (19, 20). Our findings align with these reports, with the vast majority of patients in stage III. Notably, an increased incidence of plasma cell leukemia was observed at initial diagnosis, which contributed to a high early mortality rate (15.9%); ASCT did not significantly prolong survival in these ultra-high-risk cases.

Our survival analysis identified the number of FISH abnormalities, along with creatinine, ferritin, calcium levels, and MRD status, as factors strongly correlated with survival outcomes. Notably, calcium ≥3.5 mmol/L and ferritin ≥500 ng/mL emerged as independent predictors for shortened PFS. The prognostic value of severe hypercalcemia (≥3.5 mmol/L) is consistent with the findings of Maillet et al., who reported that elevated serum calcium independently predicted shorter OS in a Mexican MM cohort (HR not directly comparable due to different cut-offs) (21). However, to our knowledge, our study is the first to identify a specific ferritin cut-off (≥500 ng/mL) as an independent risk factor exclusively within the hypercalcemic MM subgroup. This ferritin threshold is higher than the 300–400 ng/mL previously reported in unselected MM populations (17), suggesting that hypercalcemic patients may exhibit a greater inflammatory burden for ferritin to attain prognostic significance. Hypercalcemia is a primary driver of AKI in MM patients (21). The pathophysiological mechanisms include: (1) direct renal vasoconstriction leading to prerenal ischemia; (2) nephrogenic diabetes insipidus causing severe volume depletion; (3) nephrocalcinosis driving tubulointerstitial fibrosis; and (4) direct tubular epithelial toxicity resulting in cast formation and obstruction (5). Rapid correction of hypercalcemia is frequently followed by recovery of renal function (22), which highlights the interplay between calcium metabolism and renal preservation. Additionally, studies suggest that concurrent use of calcium channel blockers (CCBs) may exacerbate MM-associated hypercalcemia and AKI, further complicating the clinical picture (23–25).

In conclusion, hypercalcemia in NDMM is not merely a metabolic complication but a robust indicator of aggressive disease biology, heavy tumor burden, and an elevated risk of early mortality. Correction of serum calcium for albumin is mandatory for accurate assessment of severity. Although our findings underscore the prognostic value of calcium and ferritin levels, several limitations should be acknowledged. First, the retrospective design may introduce selection bias, and the sample size (*n* = 82) limits statistical power for subgroup analyses. Second, although the two-center design was pragmatic, it does not fully eliminate center-specific practice variations. Third, ferritin was measured only at diagnosis; serial measurements during treatment might provide additional dynamic prognostic information. Fourth, we did not account for potential confounders such as iron supplementation or blood transfusions, which can influence ferritin levels. Future large-scale, multicenter prospective cohorts with serial ferritin monitoring are warranted to validate and extend our findings.

## Data Availability

The original contributions presented in the study are included in the article/supplementary material, further inquiries can be directed to the corresponding author.
